# Intraisolate Mitochondrial Genetic Polymorphism and Gene Variants Coexpression in Arbuscular Mycorrhizal Fungi

**DOI:** 10.1093/gbe/evu275

**Published:** 2014-12-19

**Authors:** Denis Beaudet, Ivan Enrique de la Providencia, Manuel Labridy, Alice Roy-Bolduc, Laurence Daubois, Mohamed Hijri

**Affiliations:** Département de Sciences Biologiques, Institut de Recherche en Biologie Végétale, Université de Montréal, 4101 Rue Sherbrooke Est, Montréal, QC H1X 2B2, Canada

**Keywords:** arbuscular mycorrhizal fungi, mitochondria, heteroplasmy, NGS and Sanger sequencing, polymorphism, gene variants coexpression

## Abstract

Arbuscular mycorrhizal fungi (AMF) are multinucleated and coenocytic organisms, in which the extent of the intraisolate nuclear genetic variation has been a source of debate. Conversely, their mitochondrial genomes (mtDNAs) have appeared to be homogeneous within isolates in all next generation sequencing (NGS)-based studies. Although several lines of evidence have challenged mtDNA homogeneity in AMF, extensive survey to investigate intraisolate allelic diversity has not previously been undertaken. In this study, we used a conventional polymerase chain reaction -based approach on selected mitochondrial regions with a high-fidelity DNA polymerase, followed by cloning and Sanger sequencing. Two isolates of *Rhizophagus irregularis* were used, one cultivated *in vitro* for several generations (DAOM-197198) and the other recently isolated from the field (DAOM-242422). At different loci in both isolates, we found intraisolate allelic variation within the mtDNA and in a single copy nuclear marker, which highlighted the presence of several nonsynonymous mutations in protein coding genes. We confirmed that some of this variation persisted in the transcriptome, giving rise to at least four distinct *nad4* transcripts in DAOM-197198. We also detected the presence of numerous mitochondrial DNA copies within nuclear genomes (numts), providing insights to understand this important evolutionary process in AMF. Our study reveals that genetic variation in Glomeromycota is higher than what had been previously assumed and also suggests that it could have been grossly underestimated in most NGS-based AMF studies, both in mitochondrial and nuclear genomes, due to the presence of low-level mutations.

## Introduction

The success of plants in colonizing land around 450 million years ago, as well as their subsequent evolution and diversification, might have been facilitated by their interaction with the arbuscular mycorrhizal fungi (AMF) (Redecker et al. 2000; Brundrett 2002), an ancient group of root-inhabiting fungi belonging to the phylum Glomeromycota (Schüssler et al. 2001). They are considered to be obligate biotrophs, and through their interaction with plants, these fungi are rewarded with fixed carbon (Kiers et al. 2011) in exchange for specialized services (e.g. enhancing nutrient and water uptake, resistance to pathogens, etc.) that improve plant health and fitness (reviewed in Smith and Read 2008). The benefit of these services could be enhanced by the manipulation of the fungal partner genetics through nuclear segregation (Angelard and Sanders 2011).

The coenocytic nature of the AMF mycelium, the absence of a uninucleated cell stage during their lifecycle (Marleau et al. 2011) and the ability of genetically divergent isolates to fuse and exchange genetic information (Croll et al. 2009; Angelard and Sanders 2011; de la Providencia et al. 2013) have challenged the interpretation of how the intraisolate genetic variation observed in these fungi is organized and maintained over multiple generations (Sanders and Croll 2010; Ehinger et al. 2012). Two possible scenarios have been proposed: 1) homokaryosis (Pawlowska and Taylor 2004) and 2) heterokaryosis (Kuhn et al. 2001; Hijri and Sanders 2005). The homokaryotic organization implies that the nuclear polymorphism reported in these organisms is the result of orthologous allelic variants partitioned between chromosomes (i.e. polyploidy) or paralogous copies within a chromosome, while in the heterokaryotic state, different allelic variants could be evenly partitioned among distinct nuclei, or be present in a group of complementary nuclei. These scenarios might not be mutually exclusive, since the genetic variation among and within AMF isolates is likely to be a continuum between these two states, being shaped by modest rates of hyphal fusion and segregation (Bever and Wang 2005). The controversial debate over the nuclear genomic organization is in stark contrast with the consensus conclusion over mitochondrial genetic variation, which has arisen from comparison of published mitochondrial genomes (mtDNA) (Lee and Young 2009; Formey et al. 2012; Nadimi et al. 2012; Pelin et al. 2012; Beaudet, Nadimi, et al. 2013, Beaudet, Terrat, et al. 2013; de la Providencia et al. 2013). These studies have revealed intraisolate homogeneity with no apparent polymorphism (i.e. homoplasmy).

Little is known about the mitochondrial inheritance process in AMF, but the observed mitochondrial genomes homogeneity was hypothesized to be the result of effective segregation and repair mechanisms (Lee and Young 2009). A previous study showed that AMF mtDNAs migrate massively in spores during their formation (Marleau et al. 2011). In filamentous fungi, sexual crosses lead to uniparental transmission of mitochondria (Mannella et al. 1979; Lee and Taylor 1993), whereas mitochondria are biparentally inherited in budding yeast (Okamoto et al. 1998; Berger and Yaffe 2000). Recently, de la Providencia et al. (2013) demonstrated the occurrence of length-heteroplasmy (i.e. coexistence of numerous mtDNA haplotypes in the same cytoplasm) in spores formed near anastomosis regions between geographically distant *Rhizophagus irregularis in vitro* isolates. However, no information is available regarding the persistence of this heteroplasmic state. Although homoplasmy seems to be the rule rather than the exception in next generation sequencing (NGS)-based AMF studies, the coexistence of numerous mtDNA haplotypes in the same cytoplasm might be a common status in natural populations. This assumption is based on the fact that in the field, AMF constantly interact with other individuals, potentially giving rise to highly dynamic and frequent hyphal fusions. Evidence of horizontal transfer of mitochondrial genes between isolates (Beaudet, Nadimi, et al. 2013) and the occurrence of homologous recombination events between distinct mtDNA haplotypes (Beaudet, Terrat, et al. 2013) also support the occurrence of heteroplasmy in AMF.

In order to test our hypothesis and investigate the presence of intraisolate allelic variations within AMF mtDNA, we returned to the basics, using a reliable polymerase chain reaction (PCR)-based approach with a high-fidelity DNA polymerase, followed by cloning and Sanger sequencing of selected mtDNA regions. We used two *R. irregularis* isolates for this study, DAOM-197198 and DAOM-242422, the latter isolated from petroleum-polluted soil. We expected to find higher intra-isolate mtDNA allelic diversity in the isolate DAOM-242422, which has recently been isolated from the field, than in DAOM-197198, which has been cultured for more than 40 generations *in vitro*, and was previously shown to have homogeneous mtDNA (Formey et al. 2012; Nadimi et al. 2012). Surprisingly, we found intraisolate allelic variation within the mtDNA of both strains. Most interestingly, we observed mtDNA variability in the model isolate *R. irregularis* DAOM-197198 that had been overlooked by previous NGS-based studies. This variation persists at the transcriptome level, resulting in the coexpression of several distinct gene variants.

## Materials and Methods

### Fungal Cultures and Growth Conditions

Cultures of *R. irregularis* DAOM-197198 and DAOM-242422 were routinely maintained in our domestic collection in modified minimal medium solidified with 0.4% (w/v) gellan gum (Sigma, Montreal, QC) by associating spores and mycelium with Ri-T-DNA transformed chicory (*Cichorium intybus* L.) roots following the protocol described in Cranenbrouck et al. (2005). The isolate DAOM-197198 has been distributed to several laboratories worldwide and maintained under *in vitro* conditions since 1992 (Cardenas-Flores et al. 2010) and its nuclear genome has been sequenced (Tisserant et al. 2013; Lin et al. 2014). *R. irregularis* DAOM-242422 (supplementary fig. S1, see Supplementary Material online) was recently isolated in our laboratory (i.e. Autumn, 2013) following the protocol described in Cranenbrouck et al. (2005), from soil contaminated with petroleum hydrocarbons (de la Providencia IE, unpublished data). DAOM-242422 was used only in its first generation (G1) in this study. We also employed cultures of DAOM-197198 maintained by the National Mycological Herbarium of Agri-Food and Agriculture Canada in Ottawa (DAOM), the Glomeromycota *in vitro* Collection (GINCO) and Premier Tech Biotechnology (Rivière-du-loup, QC, Canada), as independent sources for controls of in-house cross-contamination. All cultures were incubated at 25°C in the dark.

### DNA Extraction

Following extraction of spores and mycelium from the gellan gel (Doner and Bécard 1991), samples were pulverized in liquid nitrogen, and total genomic DNA for both cultures was extracted using the DNeasy Plant Mini kit (Qiagen, Rockville, MD) according to the manufacturer’s instructions. DNA was stored at −20°C until further use.

### Molecular Marker Development

Multiple nucleotide sequence alignments were performed using all available *R. irregularis* mitochondrial genomes published so far. Further, three intergenic regions were selected since they are noncoding, thus are prone to accumulating mutations. These regions were chosen based on their length (*cob-nad4* [783 bp], *cox2-atp8* [962 bp] and *nad4-nad1* [747 bp]), to facilitate downstream cloning and Sanger sequencing. The mitochondrial NADH dehydrogenase subunit I (*nad1*) protein coding gene (642 bp) and the single copy nuclear 40 S ribosomal protein S2 (*rps2*) (670 bp) were used as controls for background variation rate, since the first has been shown to be homogeneous in *R. irregularis* DAOM-197198 mtDNA (Formey et al. 2012; Nadimi et al. 2012) and the latter was previously shown to be polymorphic within an isolate of *R. irregularis* (Boon 2012) (supplementary table S1 see Supplementary Material online).

### Polymerase Chain Reaction

The precloning PCR mixture was made up of 1 × high-fidelity (HF) PCR buffer, 1.5 mM MgCl_2_, 0.2 mM of each deoxynucleotide triphosphate (dNTP), 0.5 mM of each primer, 1 µl unit of Phusion high-fidelity polymerase (Agilent Technologies, Canada) and 1 µl of DNA template in a volume of 20 µl. The estimated % of PCR products having an error (i.e. DNA molecules with one error) following 35 cycles amplification with the Phusion HF DNA polymerase are shown in supplementary table S2, see Supplementary Material online. Thermal cycling parameters were as follows: Initial denaturation at 94 °C for 3 min; 35 cycles at 94 °C for 30 s, 54 °C for 25 s, 72 °C for 72 s and a final elongation at 72°C for 10 min. PCRs were performed on an Eppendorf Mastercycle ProS. PCR products were separated by electrophoresis using 1% (w/v) agarose gel, stained using Gel-Red dye (Life Technologies, Burlington, ON), visualized under ultraviolet light and images were recorded by the Gel-Doc system (Bio-Rad, Mississauga, ON).

### Cloning, Postcloning PCR, and Sequencing

PCR products were purified with the Qiaquick PCR purification kit (Qiagen, Toronto, ON) and quantified using the Qubit 2.0 fluorometer (Invitrogen, Life Technologies, Montreal, QC) according to the manufacturer’s instructions. PCR products were cloned using the StrataClone Ultra Blunt PCR Cloning Kit (Agilent Technologies, Mississauga, ON) following manufacturer’s recommendations. Aliquots of 2 µl of ligation product were incubated at room temperature for 5 min with 3 µl of StrataClone Cloning Buffer and 1 µl of StrataClone vector Mix. An aliquot of 1 µl of ligation product was used to transform one tube of competent cells. After a 45 s. heat shock at 42°C, 250 µl of SOC medium was added to the transformed cells and incubated at 37°C with agitation for 1 h. Transformed cells (100 µl) were spread on LB agar plates containing X-Gal (40 mg/ml), IPTG (100 mM), and ampicillin sodium salt (Fisher Scientific, Montreal, QC) (100 mg/ml). After an overnight incubation at 37°C, white bacterial colonies were picked and transferred into 20 µl of PCR master mix for amplification. A screening PCR protocol was performed using KAPA2G Taq (VWR, Montreal, QC) with the insert primers as follows: initial denaturation at 94°C for 3 min, followed by 35 cycles at 94°C for 30 s, 54°C for 25 s, 72°C for 72 s, and a final elongation at 72°C for 10 min. Sanger sequencing was commercially performed on an Applied Biosystems 3730xl DNA analyzer at the Genome Quebec Innovation Centre (McGill University, Montreal, QC).

### Sequence and Diversity Analysis

Sequences were edited, cleaned, and assembled using Geneious Pro version 6.1.2. (Biomatters Ltd., Auckland, New Zealand). Alignments were done using MUSCLE version 3.5 (Edgar 2004). In the presence of a putative SNP site, we verified that each base call was unambiguously supported by the trace and confirmed its occurrence if it was present on both sequencing strands. If only one sequence identified the SNP, the anomaly was considered a sequencing error. Chimeras were identified with the Bellerophon software (Huber et al. 2004) using the Huber-Hugenholtz correction parameter with a 300-bp window. All sequences were deposited in GenBank, and are registered under the accession numbers KJ775870–KJ776340.

For each selected region, contigs were imported into the program Mothur version 1.29.2 (Schloss et al. 2009) in order to compute allelic frequencies. Two sequences with a single nucleotide difference were considered distinct alleles. To provide an estimation of the allelic diversity present in each isolate and loci sampled at different depths (i.e. different number of clones), we performed a coverage-based rarefaction and extrapolation analysis using the iNEXT package version 1.0 (Hsieh 2013) in R version 3.0.2 (Team 2013) with 1,000 bootstrap resampling for the construction of 95% confidence intervals [details of this method are described in Chao et al. (2014) and Chao and Jost (2012)]. The estimated sample coverage, which is a measure of sample completeness (Good 1953), and the Chao-1 index, which is an estimator of asymptotic species richness (Chao 1984), were also computed with the iNEXT package.

### mRNA Expression Experiment

Taking into account the amount of variation we found in the *nad4* C-terminal region, we asked whether the observed variation of this particular locus persisted in the transcriptome. Four pairs of variant-specific primers, along with an intergenic control region, were designed (supplementary table S1 see Supplementary Material online) in order to perform RT-PCR confirming their mRNA expression. RNA was extracted from fresh DAOM-197198 cultures using an E.Z.N.A. fungal RNA Kit (VWR, Montreal, QC) according to the manufacturer’s recommendations. In total, 50 µl of 100 ng/µl RNA was collected and stored at −80°C until use. The SuperScript III reverse transcriptase kit (Life Technologies, Burlington, ON) was used for cDNA synthesis modifying the manufacturer’s recommendations according to Beaudet, Nadimi, et al. 2013, with oligo dt (12–18) and gene-specific primers. The resulting cDNA was stored at −20°C until use.

PCR amplifications with cDNA as a template were carried out using 1 × PCR buffer, 1.5 mM MgCl_2_, 0.2 mM of each deoxynucleotide triphosphate (dNTP), 0.5 mM of each primer, 1 µl unit of Taq Kapa biosystems (VWR, Montreal, QC), and 2 µl of cDNA template in a total volume of 20 µl. Thermal cycling parameters were as follows: initial denaturation at 95°C for 2 min, 35 cycles at 95°C for 30 s, 55°C for 30 s, 72°C for 40 s and final elongation at 72°C for 12 min. PCR reactions were performed on an Eppendorf Mastercycle ProS. (Eppendorf, Mississauga, ON). PCR products were visualized as described above but using a 2% (w/v) agarose gel.

### Searching for Mitochondrial DNA Copies (numts) in Nuclear Genomes

Based on the study by Lee and Young (2009) suggesting the presence of numts in *R. irregularis* (i.e. former *Glomus intraradices* isolate 494), a BLASTn analysis was performed on the nuclear assemblies of *R. irregularis* DAOM-197198 (Tisserant et al. 2013; Lin et al. 2014) using all the mitochondrial loci investigated in this study as the query. We considered the four single nuclei (N6, N31, N33, and N36) and two DNA samples (DNA1 and DNA2) of Lin et al. (2014) as independent assemblies, along with the one published by Tisserant et al. (2013). The presence of potential numts was considered if they met the following criteria: more than 100 nucleotides in length, with an *e*-value of 1 E−20 or higher and flanked by nonmitochondrial sequences on the contig. We then designed primers specifically targeting the nuclear contig flanking regions of the *bona fide nad4-nad1* numt in order to confirm its presence and rule out the possibility of an assembly artifact (supplementary table S1 see Supplementary Material online).

## Results

### Allelic Diversity Comparison of Two *R. irregularis* Isolates

**Fig. 1.—:**
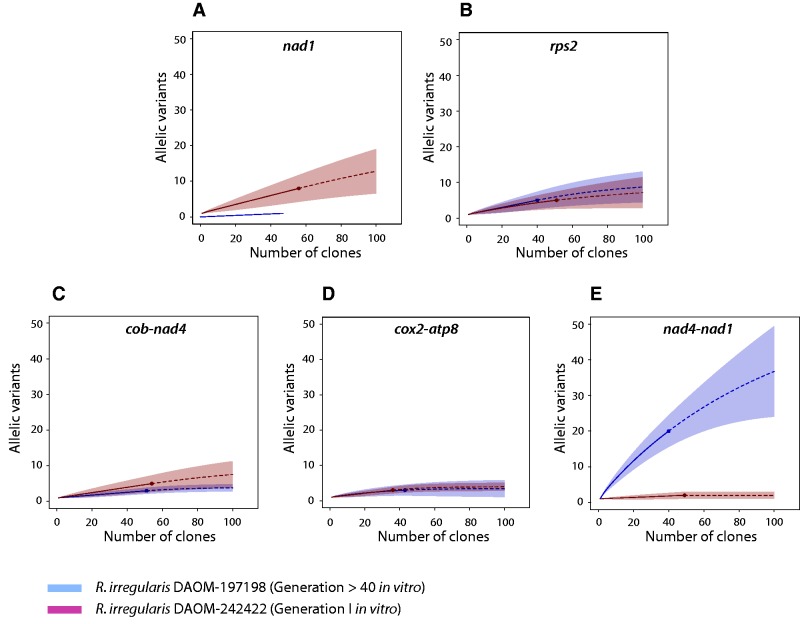
Rarefaction curves inferred with the allelic diversity present at each investigated locus. (*A*) The *nad1* gene and (*B*) the nuclear single copy *rps2* gene were used as background variation controls (shaded in gray) and compared with three mitochondrial intergenic regions. (*C*) The *cob-nad4*, (*D*) *cox2-atp8* and (*E*) *nad4-nad1* intergenic regions were investigated. The coverage based rarefaction (solid lines) and an extrapolation up to 100 clones (dashed lines) are shown. A confidence interval of 95% (shaded area, based on a bootstrap method with 1,000 replicates) of the allelic diversity from cloning sequencing (filled circles) is represented. The comparison was done with *R. irregularis* model isolate DAOM-197198 (blue line) and the first *in vitro* generation of *R. irregularis* isolate DAOM-242422 (red line).

A total of 471 assembled clone sequences were obtained by Sanger sequencing and further analyzed for the presence of chimeras and allelic variants. We did not confirm any chimeras and all sequences were retained for downstream analysis. All mitochondrial intergenic regions exhibited some degree of intraisolate genetic variation, generally between two and five different alleles in which the number of polymorphic sites varied (between one or two substitutions) (Table 1). We also noted the presence of a dominant allele in each region. The estimated coverage and shape of the rarefaction curves (fig. 1) indicated that most regions could have reached saturation for the two isolates. This indicates that the sequencing effort was sufficient to capture most of the allelic diversity present, but a higher sequencing depth would be necessary to estimate the extent of mtDNA variation with regards to the total fraction of the mitochondrial population harboring low level mutations (LLMs). The only exception was for the DAOM-197198 *nad4-nad1* region, where the coverage was only 63%. As expected, the *nad1* gene we used as a control for background variation did not show any polymorphism in the DAOM-197198 isolate. Indeed, the 47 clones sequenced for the DAOM-197198 strain displayed a unique allele. In contrast, for the same coding locus, eight distinct variants were identified for the DAOM-242422 isolate and the Chao-1 estimator indicates that the diversity could reach 29 alleles. These eight variants harbored 12 polymorphic sites, with, interestingly, 11 nonsynonymous mutations. Further, the nuclear *rps2* gene that we used as a high variation control was represented by five different alleles in both isolates, with four polymorphic sites. In both isolates, two of these variable sites were present in a small intron but two nonsynonymous mutations were present in DAOM-197198 compared with one in DAOM-242422. The estimated Chao-1 diversity inferred up to ten alleles for both isolates.

**Table 1:** Allelic diversity analysis features at all investigated loci in two *R. irregularis* isolates

Locus	Isolate DAOM/sequence length^a^	Number of clones	Number of alleles	Dominant allele frequency	Polymorphic sites^b^	Chao-1 estimator (lci-hci)^c^	Good’s coverage (%)^d^
*nad1*	197198 (514 bp)	47	1	47	0	1,00 (1,00–1,00)	100
	242422 (514 bp)	56	8	49	12 ^e^	28,62 (6,54–50,71)	88
*rps2*	197198 (306 bp)	40	5	36	4	10,85 (4,21–17,49)	90
	242422 (306 bp)	51	5	46	4	9,41 (0,92–17,89)	94
*cob-nad4*	197198 (655 bp)	51	3	49	2	3,98 (2,91–5,04)	96
	242422 (683 bp)	54	5	50	4	10,89 (4,19–17,59)	93
*cox2-atp8*	197198 (672 bp)	46	3	43	2	3,49 (0,83–6,15)	98
	242422 (680 bp)	37	3	35	2	3,97 (2,82–5,12)	95
*nad4-nad1*	197198 (541–619 bp)	40	20	16	22	56,56 (−13,76 to 126,89)	63
	242422 (619 bp)	49	2	48	1	2,00 (1,06–2,94)	98

^a^Length of the sequences analyzed at each loci, differences between or within isolates are explained by the presence of indels.

^b^Indels ≥2 nucleotides were counted as a single polymorphic site.

^c^The lower confidence interval (lci) and higher confidence interval (hci) values are the bounds on the upper and lower 95% confidence intervals for the average Chao-1 values. In other words, the observed richness was between those two numbers in 950 of the 1,000 bootstrap iterations (Chao 1984).

^d^The estimated sample coverage, which is a measure of sample completeness (Good 1953).

^e^Out of the 12 SNPs found, 11 were nonsynonymous mutations in the *nad1* CDS.

**Fig. 2.—:**
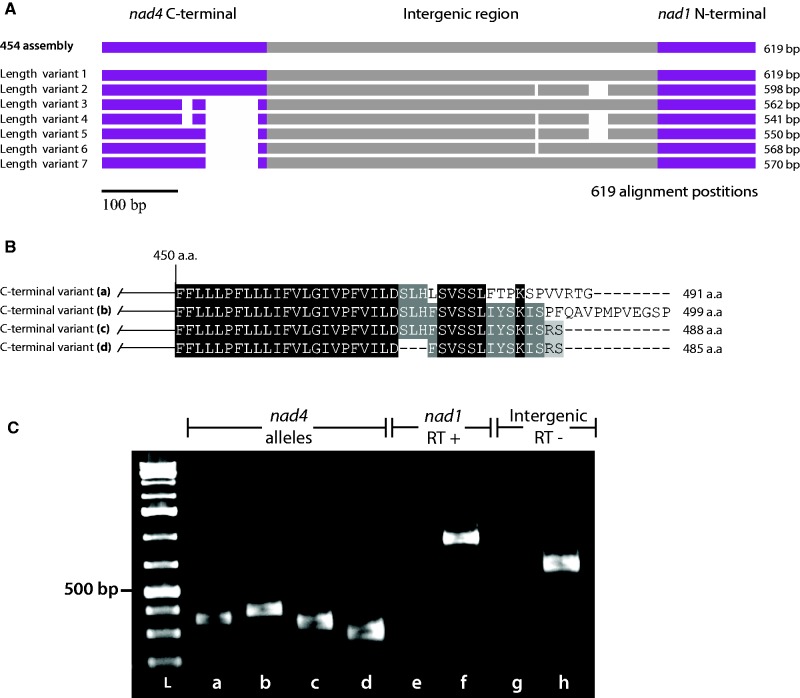
Schematic representation of the intra-isolate mitochondrial allelic diversity found in the *nad4-nad1* intergenic region in the model AMF isolate *R. irregularis* DAOM-197198. (A) The seven structural variant found in the *nad4-nad1* intergenic region are shown in a nucleotide alignment. (B) The four *nad4* C-terminal protein variants are presented in an amino acid alignment. (C) Electrophoresis gel of the RT-PCR reactions performed on *R. irregularis* DAOM-197198 cDNA showing the expression of the four *nad4* C-terminal allelic variants. (L) low range ladder, (a–d) mRNA expression of all four *nad4* length variants, (e, f) reverse transcription negative control in a mitochondrial intergenic region on cDNA and DNA templates, respectively, (g, h) reverse transcription positive control in the *nad1* gene performed on RNA and cDNA templates, respectively. Further, Sanger sequencing was used to confirm the transcripts identity.

Surprisingly, the DAOM-197198 *nad4-nad1* intergenic region exhibited the highest diversity, with 20 observed variants out of the 56 predicted *in silico* alleles. Within this region, seven structural variants showed the presence of indels (fig. 2*A* and *B*), and among these variants, four were variable in the C-terminal region of the NADH dehydrogenase subunit 4 (*nad4*) mitochondrial protein-coding gene. This observed variation in the *nad4* C-terminal region did not cause any frameshifts that could potentially impact downstream transcription, but rather introduced the possibility of four alternative stop codons. The same structural variation was not found in the DAOM-242422 isolate. Interestingly, the 454-sequencing reads of the DAOM-197198 isolate showed the presence of these indels (data not shown), which had previously been overlooked (Nadimi et al. 2012). Also, to rule out the possibility of in-house contamination, we confirmed the presence of the allelic variation, using as independent sources two other DAOM-197198 isolates originating from different laboratories (i.e. GINCO and Premier Tech) (supplementary fig. S2, see Supplementary Material online).

### Structural Allelic Variants and Their mRNA Expression

As described above, the *nad4-nad1* mitochondrial intergenic region of the model isolate *R. irregularis* DAOM-197198 exhibited four alleles variable in the C-terminal region of the *nad4* mitochondrial protein-coding gene. The assessment of the mRNA expression showed that all four variants were coexpressed, and thus gives rise to at least four putative distinct *nad4* proteins, ranging from 485 to 499 amino acids in length (fig. 2).

### Presence of Nuclear mtDNA Copies (numts)

**Fig. 3.—:**
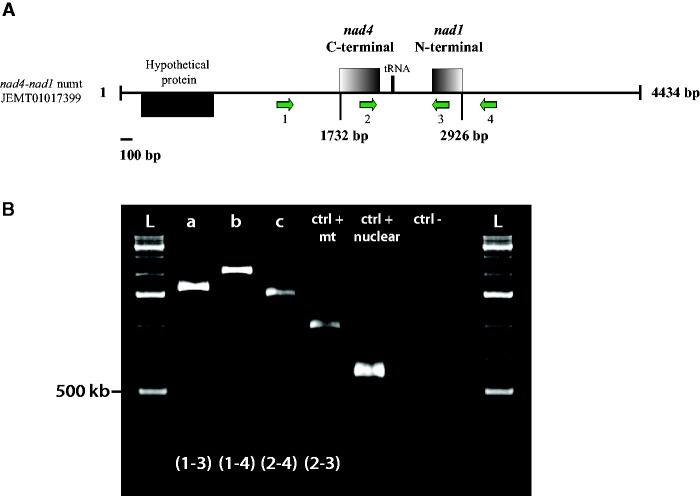
Experimental confirmation of a nuclear mitochondrial DNA (numt) copy. (A) Schematic representation of the *R. irregularis* DAOM-197198 nuclear contig harboring the putative *nad4-nad1* numt. (B) Electrophoresis gel of syntenic PCR amplifications performed on the flanking regions of the *nad4-nad1* intergenic region nuclear localization. Different combinations of primers were used in order to confirm the presence of the complete intergenic region on the nuclear contig (a–c). A mitochondrial positive control was done (ctrl + mt), with primer spanning from *nad4* to *nad1*. The nuclear positive control (ctrl + nuclear) was performed with the *rps2* primers, while the negative control (ctrl −) was done using the same primers without DNA template. All amplifications corresponded to the expected length and were further corroborated by Sanger sequencing (results not shown).

The investigation for the presence of numts in the recently published nuclear genome assemblies (Tisserant et al. 2013; Lin et al. 2014) revealed a complete *nad4-nad1* mitochondrial intergenic region, with 98% nucleotide identity to its mitochondrial counterpart. This numt was only present in one (i.e. DNA2) of the seven assemblies. We confirmed its presence by performing syntenic amplifications with primers that specifically targeted the nuclear contig flanking regions found in the Lin et al. (2014) consensus assembly (fig. 3). When we searched for the *nad4-nad1* numt flanking regions in the other assemblies, our results showed that the flanking regions are found on distinct contigs. We also detected the presence of *nad4* and *nad1* partial coding sequences (CDS), along with partial *cox2-atp8* sequences on nuclear contigs. We did not find any potential numts for the *cob-nad4* intergenic region (Table 2).

**Table 2:** BLASTn survey of all putative mtDNA mitochondrial loci investigated in this study, present in the nuclear genome assemblies of *R. irregularis* DAOM-197198

Nuclear assembly	mt-DNA copies in the nuclear genome (numts)^a^				
	*nad1*^b^	*nad4*^b^	*cox2-atp8*^b^	*cob-nad4*	*nad4-nad1*
Lin et al. (2014)^c^					
DNA1	○	JARA01006962	JARA01006115	○	○
DNA2	○	JARB01006781	JARB01003323	○	JARB01002805^d^
N31	○	JAQW01003701	JAQW01006974	○	○
N33	JAQX01002384	○	○	○
	JAQX01002367			
N36	JAQY01000696	JAQY01000696	○	○	○
		JAQY01007470			
N6	○	JAQZ01003472	JAQZ01005991	○	○
Tisserant et al. (2013)					
	○	AUPC01008815	○	○	○

^a^numts >100 nucleotides were taken into account.

^b^Only partial coding gene sequences were found in the nuclear genome.

^c^The reference genome available on GenBank, assembled based on the reads used for genome assemblies in JAQW00000000, JAQX00000000, JAQY00000000, JAQZ00000000, JARA00000000 and JARB00000000, was not used in the analysis.

^d^A bona fide complete *nad4-nad1* intergenic region found on a nuclear contig. Its presence was confirmed by syntenic PCR amplifications (supplementary fig. S4, see Supplementary Material online).

## Discussion

### Mitochondrial Genetic Diversity Is Higher than Assumed

Although, there is no unambiguous evidence of homoplasmy in Glomeromycota, it has been agreed by consensus that low (or nonexistent) variation of mtDNA is a ubiquitous and intrinsic feature of AMF (Raab et al. 2005; Börstler et al. 2008; Lee and Young 2009; Formey et al. 2012). In this study, we examined the inter- and intraisolate mtDNA variation of two *R. irregularis* isolates using cloning and Sanger sequencing approaches in selected mitochondrial regions. We have found intraisolate polymorphism in both isolates, a part of which persists at the transcriptome level. Furthermore, the detection of intraisolate polymorphism, with the presence of nonsynonymous mutations, in the single copy nuclear *rps2* gene (Boon 2012), suggests some degree of polymorphism in the nuclear genomes.

We expected to find higher intraisolate mtDNA allelic diversity in the first generation culture of the *R. irregularis* isolate DAOM-242422 that had recently been recovered from a petroleum-polluted soil, compared with the model isolate DAOM-197198 that has been cultured for more than 40 generations *in vitro* (Cardenas-Flores et al. 2010), that was previously shown to be homogeneous (Formey et al. 2012; Nadimi et al. 2012). However, the level of polymorphism was very similar between both isolates but each one having a specific hypervariable locus. The *R. irregularis* DAOM-242422 isolate did harbor eight distinct alleles for the *nad1* locus, with 11 out of 12 nonsynonymous mutations in the polymorphic sites, compared with the single homogeneous locus found in DAOM-197198. In contrast, a significant amount of variation was found in the *nad4-nad1* region for DAOM-1917198 with 20 putative alleles, compared with the DAOM-224422 isolate, where only two alleles were detected. Ours results did not allow to explain the unexpected pattern of mtDNA variation between both isolates; however, it would be interesting to study whether the differential selection pressures of the contrasting environments of origin of the two *R. irregularis* isolates might be related to the differences observed in the allelic structural variation and diversity. Such study would necessary need a full view of the mtDNA diversity since the study of few selected loci might underestimate major variability within and between isolates.

The divergence observed in the *nad4* C-terminal region in *R. irregularis* DAOM-197198 was similar to the endonuclease-mediated partial gene duplication previously reported in AMF mtDNA and other fungi (Paquin et al. 1994; Beaudet, Nadimi, et al. 2013), but we have not found an endonuclease ORF, nor any eroded remnants downstream of the sequence. The presence of these indels in the *nad4* CDS was rather surprising, since their occurrence did not hamper translation of the affected genes, but they could potentially give rise to the expression of at least four putative protein variants (fig. 3*C*). Although we did not test the mRNA expression of the eight distinct alleles for the *nad1* locus found in DAOM-242422, we hypothesized that this could also lead to the coexpression of at least eight different *nad1* protein variants in that particular isolate. It would be interesting to investigate whether the segregation or differential expression of mitochondrial variants could influence both fungal and plant fitness, as was previously demonstrated for the nuclear genome (Angelard and Sanders 2011).

### Occurrence of Mitochondrial DNA Copies in Nuclear Genomes (numts)

The presence of single-base substitutions and indels, within the same region (i.e. *nad4-nad1*), has already been reported (Lee and Young 2009), but these variants were attributed to the presence of numts. When we searched for possible numts in the assembled nuclear genome of *R. irregularis* DAOM-197198 (Tisserant et al. 2013; Lin et al. 2014), we found partial CDS of the *nad4* and *nad1* gene, with no start codons (Table 2). Surprisingly, we found a *bona fide* complete *nad4-nad1* intergenic region (with again partial CDS of each gene) on a nuclear contig. The latter was present in only one of the seven assembled nuclear genomes that we investigated. Therefore, it could have been the result of an assembly artifact, especially since the intergenic region was almost perfectly conserved, which is unexpected since a rapid erosion is usually observed for these transfers (Hazkani-Covo et al. 2010). However, we confirmed and validated its presence by syntenic amplifications with specific primers (fig. 3). It is still unclear whether its absence in the other whole genome shotgun data is due to an incomplete assembly or that it is simply not present. But since this pattern of allelic variation in the *nad4-nad1* region is not observed in the *R. irregularis* isolate DAOM-242422 investigated in that study, it suggests that numts insertions are likely to vary between isolates of the same species.

Taking these observations into account, we suggest that the observed variation might have been shaped by the ongoing evolutionary process of DNA transfer from mitochondria to the nucleus (Hazkani-Covo et al. 2010). Numts are widespread in eukaryotes, and are generally thought to be nonfunctional (Bensasson et al. 2001). The latter, combined with the presence of only partial mitochondrial CDS in the *R. irregularis* nuclear genome, testifies that their occurrence does not compromise the validity of our results, since we have demonstrated the unambiguous presence of allelic variants in the mtDNA with the confirmation of their mRNA expression, supporting intrinsic heteroplasmy. Interestingly, the presence of such a recent mtDNA to nuclei transfer means that the high allelic variation we observed in the *nad4-nad1* region might be shared not only between mt-haplotypes, but also among distinct nuclei.

### The Genetic Organization of Glomeromycota Challenges NGS-Based Studies

Previously, low levels of intraisolate mtDNA variation (i.e. polymorphism) (Lee and Young 2009) and/or high rates of short indels in reads from 454-sequencing data (Formey et al. 2012) were attributed to sequencing errors. A common challenge when working with NGS data is to distinguish true SNPs from errors generated by the NGS platforms particularly in 454 datasets, that can be due to multiple factors such as base-calling and alignment errors. Further, the downstream assembly process might purge variation within a polymorphic sample, hampering the detection of SNPs and indels (Miller et al. 2010). Also, the filtering algorithms that are commonly used to analyze allelic diversity in NGS reads appears to be a critical step. The usual filtering parameters consider a variant only if it is confirmed at least twice in a read set. However, some applications, such as the detection of heteroplasmic mutations in mtDNAs, or mutations in pooled DNA samples, require the identification of LLMs, that are present in frequencies well below what is typically sequenced in NGS studies (i.e. heterozygous or homozygous mutations) (Li and Stoneking 2012). These parameters are particularly questionable when searching for diversity in a coenocytic and multinucleated organism such as AMF, where a considerable sequencing depth might be required to confirm LLMs present in the genome population. The genomic and transcriptomic composition of individual nuclei and/or mitochondrial genomes could be concealed in the bulk NGS signal and *de novo* genome mutations easily lost in the downstream analysis (Macaulay and Voet 2014).

It is difficult to disentangle the nuclear and mitochondrial genetics of AMF, mainly because there is no stage in their life cycle where a cell harbors a single nucleus (Marleau et al. 2011). The absence of such a genetic bottleneck allows multiple nuclei and mitochondria to be inherited through generations (Ehinger et al. 2012), possibly resulting in individuals containing a variable number of diverse genomes (Boon et al. 2013). Some studies have corroborated this issue by detecting genotypic inheritance at different frequencies (i.e. segregation) of known or anonymous alleles within the progeny (Angelard and Sanders 2011; Boon et al. 2013). These studies have also suggested that genetic variation has never been exhaustively sampled for these fungi (Boon et al. 2013). The coexistence of multiple distinct nuclear and mitochondrial genomes could represent an important evolutionary feature responsible for the success of these putative asexual fungi.

### Conclusions and Outlook

We have detected intraisolate mitochondrial variation in two isolates of *R. irregularis*, a part of which persisted at the transcriptional level, as already demonstrated for some nuclear markers. These results led us to reject the hypothesis of homoplasmy, and lend support to the idea that heteroplasmy occurs in AMF. Furthermore, we showed that the presence of intraisolate mitochondrial variation is difficult to distinguish from NGS sequencing errors, but also from nuclear mtDNA copies. In light of these results, we suggest that mtDNA allelic diversity might be critically underestimated. This raises the question of whether the polymorphism detected in this study has been overlooked in previous NGS-based studies, and likely in other published mitochondrial and nuclear genomes. Future research should examine the extent of mtDNA variation with regards to the total fraction of the mitochondrial population harboring LLMs, the extent of mitochondrial transfer to the nuclear genome in AMF, and whether coexpression of mtDNA variants and their segregation might influence the AM symbiosis. These issues are of great importance for the management of agroecosystems, since the manipulation of AMF genetics have been shown to have differential effects on plant fitness.

## Supplementary Material

Supplementary tables S1 and S2 and figures S1 and S2 are available at *Genome Biology and Evolution* online (http://www.gbe.oxfordjournals.org/).

Supplementary Data
