# Supplementary material for: Intraisolate Mitochondrial Genetic Polymorphism and Gene Variants Coexpression in Arbuscular Mycorrhizal Fungi
Source: Genome Biol Evol. 2014 Dec 19;7(1):218–27. doi: 10.1093/gbe/evu275 (PMC4316628; doi:10.1093/gbe/evu275)
Supplement: Supplementary Data [file supp_7_1_218__index.html]

Intra-isolate mitochondrial genetic polymorphism and gene variants co-expression in arbuscular mycorrhizal fungi — Intraisolate Mitochondrial Genetic Polymorphism and Gene Variants Coexpression in Arbuscular Mycorrhizal Fungi — Supplementary Data 

# Intraisolate Mitochondrial Genetic Polymorphism and Gene Variants Coexpression in Arbuscular Mycorrhizal Fungi

## Supplementary Data

files

**Files in this Data Supplement:**

- Supplementary Data - docx file
- Supplementary Data - doc file
