# Supplementary material for: Intraisolate Mitochondrial Genetic Polymorphism and Gene Variants Coexpression in Arbuscular Mycorrhizal Fungi
Source: Genome Biol Evol. 2014 Dec 19;7(1):218–27. doi: 10.1093/gbe/evu275 (PMC4316628; doi:10.1093/gbe/evu275)
Supplement: Supplementary Data [file supp_evu275_New_Microsoft_Office_Word_Document.docx]

**Supporting information**

**Tables**

**Table S1**: List of primers used in this study.

**Table S2:** The estimated % of PCR products having an error (i.e. DNA molecules with 1 error) following 35 cycles amplification with the Phusion High-Fidelity DNA polymerase (HF Buffer; fidelity 4.4 x 10^-7^ error rate), compared with the % of polymorphic sites found at the different loci in both isolates investigated in this study.

**Figures**

**Figure S1**: Morphological description of *Rhizophaghus irregularis* DAOM-242422.

**Figure S2**: Validation of the occurrence of length variation in *R. irregularis* DAOM-197198 using independent isolates from different locations.
