# Supplementary material for: Intraisolate Mitochondrial Genetic Polymorphism and Gene Variants Coexpression in Arbuscular Mycorrhizal Fungi
Source: Genome Biol Evol. 2014 Dec 19;7(1):218–27. doi: 10.1093/gbe/evu275 (PMC4316628; doi:10.1093/gbe/evu275)
Supplement: Supplementary Data [file supp_evu275_Supporting_information_GBE.doc]

**Intra-isolate mitochondrial genetic polymorphism and gene variants co-expression in arbuscular mycorrhizal fungi**

Denis Beaudet^†^, Ivan Enrique de la Providencia^†^, Manuel Labridy, Alice Roy-Bolduc, Laurence Daubois and Mohamed Hijri^*^

*Institut de Recherche en Biologie Végétale, Département de Sciences Biologiques, Université de Montréal, 4101 Rue Sherbrooke Est, Montréal (Québec) H1X 2B2, Canada*

^†^ These authors contributed equally to this work

* Author for Correspondence: Mohamed Hijri, Department of biological sciences, University of Montreal, Montreal, Quebec, Canada, mohamed.hijri@umontreal.ca

**Supporting information**

**Table S1**: List of primers used in this study.

| **mt-DNA region** | **Primer** | **Sequences (5’-3’)** | **Size (bp)** |
| --- | --- | --- | --- |
| *nad1* | F  R | ATGCTATTCTTCTTACTGGAATC  CTGCTACGAGCTCTTGTTCTG | 642 |
| nuclear *rps2* | F  R | GTTCCTGTTACGAAACTTGGG  CCAGTAACCTTGCAAGGAACAG | 670 |
| *cob-nad4* | F  R | GATCTTCTGCTTTCCGACCAT  TGGACAAACTGGAAGTGGCT | 783 |
| *cox2-atp8* | F  R | TGGTGTCCTTCATTATGGTA  ACGTTGAAGCAGTTGAGGA | 962 |
| *nad4-nad1* | F  R | GGAGTCCTAGCCGTTACCTT  AAGGTTGAAGAATCCCGTAA | 747 |
| **RT-PCR** |  |  |  |
| *nad4* cDNA-specific | **F** | **TGGCAGTACCACTAACGGCTAAC** |  |
|  | S-R1 | AGCCTTCTACCGGCATAGGT | 347 |
|  | S-R2 | TTAGCCCGTTCTAACTACGG | 331 |
|  | S-R3 | TTTCTAACTACGGGAGATTTTGGA | 322 |
|  | S-R4 | GATGATACAGAAAAATCTAGGATGACG | 282 |
|  | Intergenic  Ctrl-R | CTAGATTAGCCCGTTCTAACTACG | 384 |
| **numt PCR** |  |  |  |
| *nad4-nad1* numt | F | TGCTATTACACCACATACTGCA | 1600 |
|  | R | TGGCTGATAAACTGAATAGGTGGA |  |

**Table S2:** The estimated % of PCR products having an error (i.e. DNA molecules with 1 error) following 35 cycles amplification with the Phusion High-Fidelity DNA polymerase (HF Buffer; fidelity 4.4 x 10^-7^ error rate), compared with the % of polymorphic sites found at the different loci in both isolates investigated in this study.

| **Locus** | **Expected error rate (%) *** | **% of polymorphic sites (DAOM-197198)** | **% of polymorphic**  **sites (DAOM-242422)** |
| --- | --- | --- | --- |
| *nad1* (514 bp) | 0.79 | 0.00 | 21.40 |
| *rps2* (306 bp) | 0.47 | 10.00 | 7.80 |
| *cob-nad4* (683 bp) | 1.05 | 3.90 | 7.40 |
| *cox2-atp8* (680 bp) | 1.05 | 4.30 | 5.40 |
| *nad4-nad1* (619 bp) | 0.95 | 55.00 | 2.00 |

*Based on: www.thermoscientificbio.com/webtools/fidelity/

**
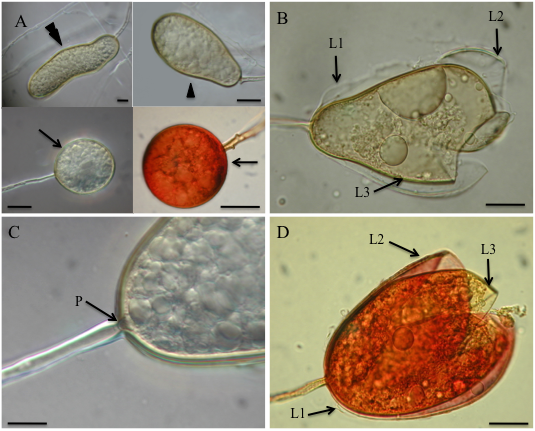
**

**Supplementary Figure 1. Morphological description of *Rhizophaghus irregularis* spores DAOM-242422 under *in vitro* conditions**. **A)** Spores are hyaline to pale yellow, mostly ovoid (black arrow) 65-70 µm to oblong (arrowhead) to irregular 65-77X96-240 µm (double arrowhead), scale bars=30 µm. **B)** The outermost layer (L1) is mucilaginous and hyaline ~ 1.5 µm thick, the second layer (L2) is rigid, smooth and hyaline ~ 1.5 and L3 is smooth and pale yellow ~ 1.5-3.2 µm. L1 and L2 were closely attached to each other forming a unique shell, easily detachable for the third layer (L3) scale bar=20 µm. **C)** The subtending hyphae (6.5-8 µm) was hyaline and straight, the pore was wide open at the spore-base but sometimes occluded by a funnel-shaped like to curved septum (P), scale bar=15 µm **D)** Spores in Melzer’s reagent stained from red-violet in L1-L2 to brown in L3, scale bar=20 µm.

**Supplementary Figure 2. Confirmation of the occurrence of length variation in the nad4 C-terminal region of the model *R. irregularis* isolate DAOM-197198 originating from three different locations. A)** The seven structural variants found in the *nad4-nad1* intergenic region are shown in an alignment with the primers designed to confirmed length variation in the different samples (green arrows). **B)** (a) Electrophoresis gel of the PCR reactions performed on *R. irregularis* DAOM-197198 originating from our laboratory located in Montreal-QC-Canada, (b) PremierTech biotechnology located in Rivière-du-loup-QC-Canada and (c) Agrifood Canada located in Ottawa-ON-Canada, respectively, along with (d) the negative PCR control. All DAOM-197198 isolates showed the presence of double bands at the investigated locus, thus confirming the occurrence of length variation in all strains and rule out the possibility of in-house contamination.
